# Descriptive analysis of sepsis in a developing country

**DOI:** 10.1186/s12245-015-0068-1

**Published:** 2015-06-06

**Authors:** Gilbert Abou Dagher, Mothana Saadeldine, Rana Bachir, Dina Zebian, Ralphe Bou Chebl

**Affiliations:** Department of Emergency Medicine, American University of Beirut, Beirut, Lebanon

## Abstract

**Background:**

Most studies on sepsis were conducted in developed countries. The aim of this study is to report on a series of patients with sepsis in a tertiary hospital in a developing country.

**Methods:**

Patients admitted through the emergency department of a single university-based institution between January 2008 and June 2012, with a final diagnosis of sepsis, bacteremia, or septic shock, were retrieved. A sample of 97 patients was selected. Vital signs at presentation, number of SIRS criteria, use of vasopressors and steroids, and in-hospital mortality were recorded.

**Results:**

The mean age was 70.09 ± 16.82, ranging from 19 to 96 years; 48.5 % were females and 51.5 % were males; 42.3 % of the patients were found to be bacteremic. IV fluid requirement during the first 6 h was 1.75 ± 1.96 l. The time for antibiotic initiation was 3.43 ± 4.48 h, with 87.6 % of the antibiotics initiated in the emergency department. Norepinephrine was the most commonly used vasopressor (38.1 %) followed by dopamine (8.2 %), and the inotrope dobutamine (4.1 %); 45.3 % of the patients were admitted to the intensive care unit (ICU), and the remaining 54.7 % were managed on the general practice unit (GPU). A total of 30 (30.9 %) septic patients died. The 28-day mortality was 20.6 %. Deceased patients had greater vasopressor use, a longer stay in the ICU (*p* = 0.001), and a longer time to norepinephrine use (*p* = 0.004).

**Conclusions:**

This is the first study providing an in-depth analysis of sepsis patients in a developing country, looking at in-hospital mortality, SIRS criteria utility, and at the overall sepsis management.

## Background

Sepsis has gained worldwide recognition in the last two decades. The Early Goal-Directed Therapy (EGDT) study introduced protocol-based care in sepsis management in 2001 [1], followed by the Surviving Sepsis Campaign (SSC) in 2002 [2], to the NIH-funded PROCESS and ARISE trials that looked at the value of such protocol-based intervention [3, 4]. Despite the enormous amount of sepsis research, the incidence of affected patients in the USA has doubled in the past decade, and approximately 750,000 persons are affected annually [5], with one septic patient presenting to an emergency department in the USA every minute [6]. Most sepsis studies, whether evaluating the overall mortality of the disease or the benefit of a specific treatment modality, were done in developed countries such as the USA, Australia, and Europe. There are no studies looking at sepsis, the value of SIRS criteria, or its related mortality in a developing country. The aim of this study is to describe the pathogens, underlying medical conditions, management, and mortality of patients presenting with sepsis at the American University of Beirut Medical Center (AUBMC), a 420-bed teaching hospital in Lebanon.

## Methods

A separate study at our institution looked at sepsis in dialysis patients and looked at their in-hospital mortality. There were a total of 97 end-stage renal disease (ESRD) patients. The non-ESRD patient control group was significantly larger. A convenience sample of 97 patients admitted through the Emergency Department of a single university-based institution between January 2008 and June 2012, with a final diagnosis of sepsis, bacteremia, or septic shock, was randomly selected with the help of a computer-generated program. Exclusion criteria included age less than 18 years, pregnant, and trauma cases. After an Institutional Review Board (IRB) approval from the American University of Beirut, we studied that same cohort of patients and looked at a variety of parameters related to their emergency department (ED) management, disposition, and outcome.

Age, gender, ethnicity, and history of comorbidities were obtained from the patients’ medical record. Patients’ vital signs and number of SIRS criteria were collected at initial presentation to the emergency department. The site of infection, causative microorganism, and presence of bacteremia were retrieved as well as complete blood count, electrolytes, lactate, cardiac enzymes, arterial blood gas results, and coagulation profile results. Time to antibiotics and amount of fluid resuscitation within the first 6 and 24 h, duration and type of vasopressors, and steroids administration were also noted. Disposition from the ED, length of stay in ED, intensive care unit (ICU), or general floor, was calculated. We also looked at hospital mortality as well as 28-day mortality.

### Statistical analysis

A two-tailed sample *t* test compared lengths of stay in ED, ICU, or floor; overall length of stay in hospital; time to and duration of vasopressors, antibiotics, and steroids; fluid replacement at 6 and 24 h, and vital signs at presentation and after 6 h, between deceased patients and non-deceased. A Pearson’s chi-square test was used to compare differences in distribution of bacteremia, ED disposition, use of vasopressors or steroids, and number of SIRS criteria at presentation between deceased patients and non-deceased. Statistical analyses were performed using SPSS Statistics for Windows Version 21.0. (Armonk, NY: IBM Corp).

## Results

### Patient characteristics

The mean age was 70.09 ± 16.82, ranging from 19 to 96 years; 48.5 % were females and 51.5 % were males. The most common underlying comorbidity was hypertension (58.8 %) followed by diabetes (34.0 %) and coronary artery disease (25.8 %) (Table 1). There was no significant difference in comorbidity distribution between patients presenting with a systolic blood pressure less or more than 90 mmHg. Systolic blood pressure upon presentation to the emergency department ranged from 53 to 180 mmHg, while it ranged from 66 to 177 mmHg after 6 h of presentation.

**Table 1:** Baseline characteristics of the patients

Age (years)	70.09 ± 16.8
Sex (%)	
Female	48.5
Male	51.5
Chronic coexisting conditions (%)	
Hypertension	58.8
Diabetes	34
Coronary artery disease	25.8
Congestive heart failure	16.9
Chronic obstructive lung disease or emphysema	10.3
Neurologic disease	3.1
SIRS criteria at presentation (%)	
2 or more	67
Less than 2	30
Vital signs upon presentation	
Temperature (°C)	37.55 ± 1.127
Systolic blood pressure (mmHg)	111.23 ± 27.63
Diastolic blood pressure (mmHg)	60.81 ± 16.91
Mean arterial pressure (mmHg)	77.27 ± 18.96
Heart rate (beats/min)	100.43 ± 28.67
Respiratory rate (respiration/min)	22.31 ± 6.25
Oxygen saturation (%)	94.07 ± 6.7
Baseline laboratory values	
White cell count (per mm^3^)	19.5 ± 3.34 × 10,000
Hemoglobin	10.69 ± 2.45
Hematocrit	32.27 ± 8.43
Lactate (mmol/l)	3.80 ± 3.531
Creatinine (mg/dl)	1.86 ± 1.9
Blood urea nitrogen (mg/dl)	37.03 ± 26.29
Total bilirubin (mg/dl)	3.08 ± 5.2
Partial pressure of carbon dioxide (mmHg)	36.42 ± 10.8
pH arterial blood	7.36 ± 0.13
PaO_2_/FiO_2_	241.8 ± 111.54
Bacteremia	
Yes	41
No	56
Site of infection (%)	
Urine	40.2
Lung	19.6
Unknown	19.6
Skin	10.3
GI	8.2
Endocarditis	1
Oral flora	1

### Microbiology

Bacteremia was defined as a single positive blood culture showing non-skin flora pathogens or a minimum of two positive blood culture bottles with skin flora pathogens; 42.3 % of the patients were found to be bacteremic. The most common sites of infection were genitourinary (40.2 %) followed by pulmonary (19.6 %) and integumentary (10.3 %). In 19.6 % of cases, no focus of infection could be identified (Table 1). In 76 out of 97 patients (78.4 %), a microorganism was identified with Gram-negative organisms exceeding the Gram-positives. Among the Gram-negative organisms, *Escherichia coli* has been consistently the most commonly isolated organism (38.1 %) followed by *Klebsiella* (11.3 %) and *Pseudomonas* (7.2 %). In the Gram-positive bacteremia group, coagulase-negative staphylococci represented the majority of the isolates (8.2 %) (Table 2). When stratified according to blood pressure, 38.5 % of patients with systolic blood pressure less than 90 mmHg were bacteremic in contrast to 43.5 % of patients with a systolic blood pressure greater than 90 mmHg.

**Table 2:** Causative microorganisms

Microbiology	*N* (%)^a^
*Escherichia coli*	37 (38.1)
*Klebsiella pneumoniae*	11 (11.3)
Coagulase-negative staphylococci	8 (8.2)
*Pseudomonas aeruginosa*	7 (7.2)
*Enterococcus* species	6 (6.2)
*Candida* species	6 (6.2)
Non-albicans *Candida*	4 (4.1)
*Proteus mirabilis*	3 (3.1)
Diphtheroids species	3 (3.1)
*Acinetobacter baumannii*	2 (2.1)
*Streptococcus pneumoniae*	2 (2.1)
Others *Clostridium* species, *Listeria monocytogenes*, *Morganella morganii*, *Brucella* species, *Providencia alcaligenes*	11 (11.3)

^a^More than one organism may be retrieved from a single subject

### Management

IV fluid requirement during the first 6 h was 1.75 ± 1.96 l and 3.37 ± 2.85 during the first 24 h. On the other hand, the time for antibiotic initiation was 3.43 ± 4.48 h, with 87.6 % of the antibiotics initiated in the emergency department (Table 3).

**Table 3:** Patient management characteristics

	Number	Mean ± SD	Range
LOS in ED (hours)	97	13.35 ± 17.154	1.00–137.50
LOS in ICU (days)	43	12.04 ± 13.951	0.40–70.67
LOS in GPU (days)	52	7.27 ± 5.941	0.33–30.95
LOS in the hospital (days)	97	13.86 ± 13.618	0.507–70.917
Time to vasopressors in the first 24 h (hours)	21	9.14 ± 8.027	0.50–24.00
Time to norepinephrine (hours)^a^	36	148.20 ± 282.190	1–1464.00
Time to dopamine (hours)^a^	7	278.31 ± 393.557	1–870.50
Time to dobutamine (hours)	4	59.50 ± 41.026	22–114.50
Vasopressors Tx duration (days) for those who took vasopressors in the first 24 h	21	3.33 ± 3.411	0.13–12.88
Steroid Tx duration (days)	47	7.55 ± 7.540	0.50–35.00
Time to antibiotic treatment initiation (hours)^a^	96	3.43 ± 4.479	0.167–36.33
IV fluid requirement first 6 h (liters)	96	1.75 ± 1.964	0.02–11.06
IV fluid requirement first 24 h (liters)	96	3.37 ± 2.846	0.160–16.642

^a^The time to norepinephrine, to dopamine, and to antibiotic treatment for one patient is missing since the paper containing this information was not found in the patient’s chart

The time to initiate vasopressors was 9.14 ± 8.03 h. Norepinephrine was the most commonly used vasopressor (38.1 %) followed by dopamine (8.2 %) and the inotrope dobutamine (4.1 %). Vasopressor treatment duration in the first 24 h was 3.33 ± 3.411 h. Furthermore, the hypotensive group at presentation required more vasopressors than those who presented with systolic BP ≥90 mmHg.

There was no difference in SIRS criteria between the hypotensive and normotensive group as 69 % of patients in both groups had greater than 2 SIRS criteria.

### Disposition

Admitted to the ICU were 45.3 % of the patients, and the remaining 54.7 % were managed on the general practice unit (GPU) (Table 4). The mean length of stay in the ED was 13.35 ± 17.15 h, while it was 12.04 ± 13.95 and 7.27 ± 5.94 days in the ICU and GPU, respectively. The mean length of stay in the hospital was 13.86 ± 13.62 days (Table 3); 73.1 % of patients with initial systolic blood pressure less than 90 mmHg were admitted to the ICU.

**Table 4:** Disposition of septic patients

	Number	Percent
Admission^a^		
ICU	43	45.3
GPU	52	54.7
Hospital mortality	30	30.9
Discharge home	67	69.1
28-day mortality		
Yes	20	20.6
Unknown	19	19.6

^a^Two patients have not been admitted as one of them left AMA and the other one died in the ED

### Mortality

A total of 30 (30.9 %) septic patients died. The 28-day mortality was 20.6 % with 19.6 % lost to follow-up. There was no statistically significant difference in hospital mortality between the hypotensive and normotensive groups. There was no significant difference in mean age, gender distribution, and comorbidities between the discharged and deceased group. The percentage of bacteremia was significantly higher in the survival group (92.7 %) than in the deceased group (7.3 %).

Deceased patients had greater vasopressor use and a longer stay in the ICU (*p* = 0.001). Time to norepinephrine was significantly longer in the deceased group (*p* = 0.004) (Table 5).

**Table 5:** Patient mortality characteristics

	Mortality (no)		Mortality (yes)		*p* value
	*N*	%	*N*	%	
Norepinephrine	17	45.9	20	54.1	<0.001
Dopamine	3	37.5	5	62.5	0.044
Steroid use	25	53.2	22	46.8	0.001
Antibiotics initiated in ED	61	71.8	24	28.2	0.127
Antibiotics initiated in ICU	2	33.3	4	66.7	0.248
Antibiotics initiated in GPU	4	53.2	2	46.8	0.248
SIRS					
0 or 1	22	73.3	8	26.7	0.543
≥ 2	45	67.2	22	32.8	
Bacteremia					
No	29	51.8	27	48.2	<0.001
Yes	38	92.7	3	7.3	
ICU admission	24	55.8	19	44.2	0.012
GPU admission	42	80.8	10	19.2	0.007

Vital signs upon presentation and after 6 h, IV fluids requirements during the first 6 and 24 h, and time to initiation of antibiotics were not different between the two groups. Out of 30 patients who presented initially with less than 2 SIRS criteria, 8 died during their hospital stay (26.7 %) (Table 5). Laboratory findings also were not significantly different between the two groups.

## Discussion

Since EGDT was published, there has been a steady decline in sepsis-related mortality. The rate of hospitalizations however has doubled during the past decade in the USA [7]. Although much of the therapy for severe sepsis occurs in ICU, as many as 500,000 cases of severe sepsis are initially managed in EDs annually, with an average ED length of stay of 5 h [5, 6, 8]. Recent research has shown that the most important and the cornerstone of sepsis therapy is early recognition, aggressive fluid hydration and early antibiotics [3, 4]. The surviving sepsis campaign recommends a 30 cc/kg bolus in the first 3 h of resuscitation [2]. Further fluid therapy is guided by monitoring various hemodynamic metrics such as the central venous pressure or the mean arterial pressure. The mean amount of fluids given in our cohort within the first 6 h was 1.75 l. There was however a statistically significant difference in fluid resuscitation between the hypotensive and normotensive group. This discrepancy is probably stemming from the prejudice that hypotensive patients are sicker. However, EGDT and SSC recommend aggressive hydration to both severe sepsis and septic shock regardless of blood pressure on presentation. Early antibiotics have been shown to improve survival in septic patients [9]. The mean time to antibiotic in our study was 3.43 h with the majority (87.6 %) of antibiotics started in the emergency department. It is important to note that at the time the study was conducted, our institution did not follow yet a standardized protocol-based approach to sepsis management, which would explain why most sepsis resuscitation goals were not met.

According to the literature, the most common infection sites in septic patients are the respiratory and genitourinary systems as well intra-abdominal surgical infections and indwelling catheters [5]. The most common focus of infection in our population was the genitourinary system with Gram-negative organisms being the most predominant culprits. This is in accordance with a previous study done in our institution looking at bacteremia in febrile neutropenia patients that showed the growing incidence of Gram-negative organisms [10].

Septic shock is one of the leaders in mortality nowadays, but with early recognition and treatment, we have seen an improvement in septic shock mortality [2]. The mortality from septic shock was found to be as high as 46 % in the original EGDT control group, but the implementation of protocols aiming at early identification and aggressive care has lead to an improved survival, and nowadays the mortality from septic shock ranges between 20 and 30 % [1, 2, 11]. To the best of our knowledge, there is one study that looked at sepsis and its toll in the Middle East. Memon et al. looked at the impact of a 6-h resuscitation bundle on sepsis outcome in a Saudi Arabian intensive care unit and found a reduction in mortality in septic patients from 31 to 21 % [12]. In our cohort, the hospital mortality was 30.9 % and the 28-day mortality was 20.6 %. It was interesting to note that there was no significant mortality difference between the normotensive and hypotensive patients at presentation.

There are a limited amount of ICU beds at our institution. Very often, patients requiring ICU stay tend to remain in the ED for a prolonged time until a bed is available. Should these patients improve during their ED stay, they are downgraded to a GPU bed. This in part explains our long ED length of stay and our high GPU admission rate. Our GPU beds are not equipped with continuous monitoring. Vital signs are taken every 4–6 h. Vasopressors, antibiotics, and non-invasive ventilation can be initiated on the GPU. Further invasive hemodynamic monitoring (CVP, arterial lines) occurs in the critical care units. Of all the patients who were admitted to the GPU, 10 passed away. They were stepped up to an ICU bed and eight of them were successfully moved to the MICU. The two remaining patients passed away on the wards after their families requested to make them comfort care. Overall, patients admitted to the ICU had a greater mortality, and this can be explained by the fact that sicker patients are usually admitted to critical care units.

The SIRS criteria were initially proposed as a screening method to rapidly flag possible septic patients, as sepsis is defined as having 2 or more SIRS criteria in the setting of a presumed or documented infection. Even though they were sensitive, SIRS criteria were not specific and did not correlate with mortality [13]. Owing to the fact that our study was a retrospective one, we selected patients based on their discharge diagnosis and wanted to look at the value of the SIRS criteria at presentation. It is interesting that in our study, 27 % of patients who presented with less than 2 SIRS criteria died within their hospital stay. Emergency physicians should be aware of this and should maintain a high level of suspicion when caring for septic patients presenting with normal vital signs.

### Limitations

This is a retrospective study done in a single emergency department, and as such, our study has many limitations. Some of the patients did not have repeat vitals at 6 h, and lactate was not drawn on all patients as our institution began measuring lactate on septic patients fairly recently. Our sample size is small and there was a significant amount of patients who were lost to follow-up. Furthermore, information regarding initial ED disposition or whether patients were stepped up to an ICU level bed was not available. This can be explained by our institution’s use of paper-based medical records at the time of the study, which might have lead to the loss of some patient information.

## Conclusions

This is the first study looking at an in-depth analysis of sepsis in the Middle Eastern population and examining the overall management of septic patients and their in-hospital mortality in a single tertiary care center in Beirut, Lebanon, prior to the initiation of a protocol-based approach. Although our mortality rate was in the range of the Western world, it will be very interesting to see the effect of sepsis treatment bundles advocating for early recognition, aggressive hydration, and early antibiotics on sepsis-related mortality.

We hope that it will stimulate further prospective studies on sepsis in the Middle East and other developing countries with limited healthcare resources.
